# Exposure to Wood Smoke and Associated Health Effects in Sub-Saharan Africa: A Systematic Review

**DOI:** 10.5334/aogh.2725

**Published:** 2020-03-20

**Authors:** Onyinyechi Bede-Ojimadu, Orish Ebere Orisakwe

**Affiliations:** 1 Department of Chemical Pathology, Faculty of Medicine, Nnamdi Azikiwe University, Nnewi Campus, NG; 2 Department of Experimental Pharmacology and Toxicology, Faculty of Pharmacy, University of Port-Harcourt, Rivers State, NG; 3 World Bank Africa Centre of Excellence in Public Health and Toxicological Research (PUTOR), University of Port Harcourt, Port Harcourt, Rivers State, NG

## Abstract

**Background:**

Observational studies suggest that exposure to wood smoke is associated with a variety of adverse health effects in humans.

**Objective:**

We aimed to summarise evidence from sub-Saharan Africa on levels of exposure to pollutants in wood smoke and the association between such exposures and adverse health outcomes.

**Methods:**

PubMed and Google scholar databases were searched for original articles reporting personal exposure levels to pollutants or health outcomes associated with wood smoke exposure in Sub-Saharan African population.

**Results:**

Mean personal PM_2.5_ and carbon monoxide levels in the studies ranged from 26.3 ± 1.48 μg/m^3^ to 1574 ± 287μg/m^3^ and from 0.64 ± 2.12 ppm to 22 ± 2.4 ppm, respectively. All the reported personal PM_2.5_ exposure levels were higher than the World Health Organization’s Air Quality Guideline (AQG) for 24-hour mean exposure. Use of wood fuels in domestic cooking is the major source of wood smoke exposure in this population. Occupational exposure to wood smoke included the use of wood fuels in bakery, fish drying, cassava processing and charcoal production. Females were exposed to higher levels of these pollutants than males of the same age range. Major determinants for higher exposure to wood smoke in SSA included use of unprocessed firewood, female gender and occupational exposure. We recorded strong and consistent associations between exposure to wood smoke and respiratory diseases including acute respiratory illness and impaired lung function. Positive associations were reported for increased blood pressure, low birth weight, oesophageal cancer, sick building syndrome, non-syndromic cleft lip and/or cleft palate and under-five mortality.

**Conclusion:**

There is high level of exposure to wood smoke in SSA and this exposure is associated with a number of adverse health effects. There is urgent need for aggressive programs to reduce wood smoke exposure in this population.

## Introduction

Wood smoke is a complex mixture of gases, liquids and solid particles (aerosol) produced by incomplete combustion or pyrolysis of wood and other wood products such as charcoal, wood pellets, sawdust, and so on, at elevated temperatures and reduced oxygen [1]. While complete combustion of wood requires adequate supply of oxygen and produces carbon dioxide and water with no visible smoke, incomplete combustion results in the production of smoke. Besides the major combustion products (carbon dioxide and water), wood smoke consists of more than 200 distinct organic compounds [2], many of which have been shown to induce acute or chronic health effects in exposed humans. Of these, particulate matter, especially the fine particulate matter (PM_2.5_), is of most concern. Other hazardous components of wood smoke are carbon monoxide, nitrous oxides, formaldehyde, and polycyclic aromatic hydrocarbons (PAHs), including carcinogens such as benzo(a)pyrene [2,3].

Human exposure to wood smoke is as old as mankind. In prehistoric times, man used wood as the primary fuel for heating in the cold, lighting in the dark, and for cooking food [3] and for thousands of years, wood served as the sole source of energy for humankind [4]. Although increasing modernisation has led to the supplementation of wood by fossil fuels (such as coal and petroleum products) and electricity, it is still a major source of energy for the population in developing countries accounting for 50 to 90% of the fuel used for cooking and heating purposes in this population [4,5]. The demand and use of wood fuels has also increased (especially among the poor) in many developed nations due to scarcity of fossil fuels coupled with an increasing interest in sustainable energy production [4,6].

Although there has been a decline in the proportion of the world’s households relying mainly on solid fuels for cooking, with about 60 percent of the world’s population currently using modern fuels [7], the population in sub-Saharan Africa has not kept up with this global trend as reports show that this population still has the most widespread use of solid fuels [8]. About 83% of the population in WHO African region were estimated to be primarily reliant on polluting cooking options [9]. Of all the solid fuels (wood, coal, charcoal, dung, crop residues), wood fuels (firewood, charcoal and other crop residues) are predominantly used among the population in SSA, accounting for more than 90% of residential energy consumption in rural areas [10]. It has been reported that per capita consumption of wood fuel in SSA is 2–3 times higher than that in any other region [11]. Of the 2.770 billion people in developing countries projected to depend on wood fuel by 2030, sub-Saharan Africa alone accounts for 33.14% (918 million people) of this population [12].

Indoor air pollution from inefficient traditional wood burning stoves and open fires remains the major source of wood smoke exposure to humans, especially households in rural areas of developing countries who rely exclusively on woods for their cooking and heating needs [2]. Women, who do most of the household cooking, children under the age of five and the elderly who spend more time in the household are more exposed [13,14]. Higher level of exposure may be seen in individuals with some occupations. These include wild land fire fighters [15], charcoal producers [16,17,18], farmers involved in agricultural burnings [2] and individuals involved in commercial cooking and food processing using wood fuel [19,20,21]. Other significant sources of exposure may include bushfires, consumption of food items preserved or processed with wood smoke [19,22,23] and the ambient air [24,25].

Wood fuel is cheap, has widespread availability and potential renewability [11] and as compared to fossil fuels, might help to reduce impacts of long-term carbon emissions on climate change [26]. However, exposure to smoke from its combustion has been of significant public health concern especially in developing countries. Household air pollution (HAP) from solid fuels globally accounted for 2.576 (2.216–2.969) million deaths and 77.16135 (66.08637–88.04887) million disability-adjusted life years (DALYs) in the year 2016 [27], amounting to 7.87% and 7.14% of total deaths and DALYs, respectively, attributable to all risk factors in 2016. With these figures, HAP was ranked as the 8th leading mortality risk factor and the 10th leading risk factor for disability-adjusted life-years (DALYs), globally in 2016 [27]. Low- and middle-income countries in SSA had the highest number (134) of age-standardized deaths (globally) per 100,000 capita from HAP in 2016 [9]. In 2017, 24% of global deaths and 34% of global DALYs attributable to household air pollution occurred in sub-Saharan Africa [28]. HAP from solid fuels accounted for about 35.64 % and 36.33% of total deaths caused by lower respiratory tract infections in sub-Saharan African children <5 years and women between aged 15–49 years, respectively [29].

Over the past decade, there has been a number of reviews focussing on household solid fuel use and its effect on human health [30,31,32]. However, there remains a lack of solid evidence on wood smoke exposure and the associated health effects in sub-Saharan African population. Hence, this review aims to systematically assess available evidence on exposure to pollutants in wood smoke and the health effects associated with this exposure in sub-Saharan African population. The findings of this review may help prioritize methods to control emissions from wood burning and reduce its associated diseases across sub-Saharan Africa.

## Methods

### Search strategy

A systematic literature search was done using PubMed and Google scholar databases for papers published in peer-reviewed journals from inception to December 31, 2018. To ensure the widest possible coverage of papers, we searched the databases using broad search terms, after which irrelevant papers were excluded. The following search terms were used: (‘wood smoke’ OR ‘woodsmoke’ OR ‘firewood’ OR ‘charcoal’ OR ‘solid fuel’ OR ‘indoor air pollution’ OR ‘household pollutants’ OR ‘biomass fuel’ OR ‘wildfire’ OR ‘open fire’ OR ‘domestic fuel’ OR ‘traditional stove’ OR ‘particulate matter’ OR ‘PM_10_’ OR ‘PM_2.5_’ OR ‘fire place’ OR ‘wood burning’ OR ‘cooking’ OR ‘heating’) AND (‘Names of each country in Sub-Saharan Africa’). The same terms were used for the two databases and papers were limited to those published in English language. No limitations were set for participants’ age and sex. The search was done in December, 2018 and updated in January, 2019. Reference lists of relevant reviews and included studies were also assessed for additional relevant studies.

### Study Selection

Epidemiological studies that measured personal exposure to pollutants in wood smoke, examined health effects associated with wood smoke exposure or identified wood smoke as a risk factor to any measured health effect in humans living in sub-Saharan Africa were included.

#### Inclusion criteria

The population of interest were those living in SSA irrespective of age, sex, country. We considered environmental exposure to wood smoke in household cooking and heating as well as occupational exposure to wood smoke as seen in charcoal production, commercial food processing and preparation, agricultural burning and wildfire fighting. Only studies that reported specific exposure to smoke from wood fuels (firewood, charcoal) were included. Different comparators were considered in this review. Studies that compared wood fuel users and users of liquefied petroleum gas or electricity, those that compared exposure between firewood and charcoal users or between traditional open fire and improved wood stove users, or made comparison between individuals occupationally exposed and those not exposed to wood smoke were all included in the study. Two outcomes of interest were assessed: exposure levels and health outcomes. For the exposure levels, studies that measured personal exposure levels to pollutants in wood smoke (such as personal PM_10_, PM_2.5_ or CO measurements) or any biomarker of wood smoke exposure were included. For the health outcomes, papers that reported associations between exposure to wood smoke and any health outcome in any population group living in sub-Saharan Africa were included. The study designs considered were both observational (cohort, case-control, cross-sectional studies) and experimental (randomized controlled trials) studies.

#### Exclusion criteria

Studies were excluded if they gave reports from countries other than those in SSA, reported exposure to other solid fuels or biomass fuels (such as coal or animal dung) than wood, reported exposure to biomass or solid fuel without specifying exposure to wood smoke. Studies conducted in multiple countries including those in SSA were excluded except those that gave specific data for the SSA Country. Studies without proper comparators, those that compared “clean fuels” to “dirty fuels” and those that compared two types of improved wood stoves were excluded. Studies that measured air quality as proxy to individual exposure to PM_2.5_ PM_10_ or CO as well as animal studies were excluded.

### Data Extraction

All titles, abstracts and full texts were screened according to the inclusion and exclusion criteria and data extraction forms was used to extract relevant data from the included papers. Paper eligibility was assessed independently by two reviewers and disagreements resolved by consensus. Generally, data extracted included information on: author, year of publication, country of study, study setting, study design, population of study, sample size, exposure assessment, outcome and outcome assessment method, and health effect (with the effect estimate and the associated 95% CI, where available).

### Quality Assessment

The articles with evidence on health outcomes that met the inclusion criteria were assessed for quality using a 7-point score designed by the authors, specifically for this review. The following study characteristics were used for the quality assessment and a score of 0 or 1 was given depending on whether a study met a particular characteristic or not: description of study design (papers were scored 1 or 0, depending on whether the study design was adequately described or not), sampling strategy (studies with random selection were considered better and scored 1, as randomisation is an effective safeguard against selection bias), representativeness of study population (the description of the study population was assessed and papers were scored 1 or 0 respectively, depending on whether the study subjects were representative of the population or not), ascertainment of exposure to wood smoke (papers were scored 1 or 0, depending on whether exposure was adequately measured or assessed through questionnaire report), ascertainment of health outcome (papers were scored 1 if the outcome was based on clinical diagnosis or assessed through standard protocol; and 0 if assessed through questionnaire report), selection of non-exposed controls (papers were scored 1 or 0, depending on whether or not wood smoke exposure was ascertained in these individuals) and appropriate method to control for confounding (whether adjustments were made for variable such as age, smoking status and exposure to environmental or second-hand smoke etc.).

## Results

**Figure 1:**
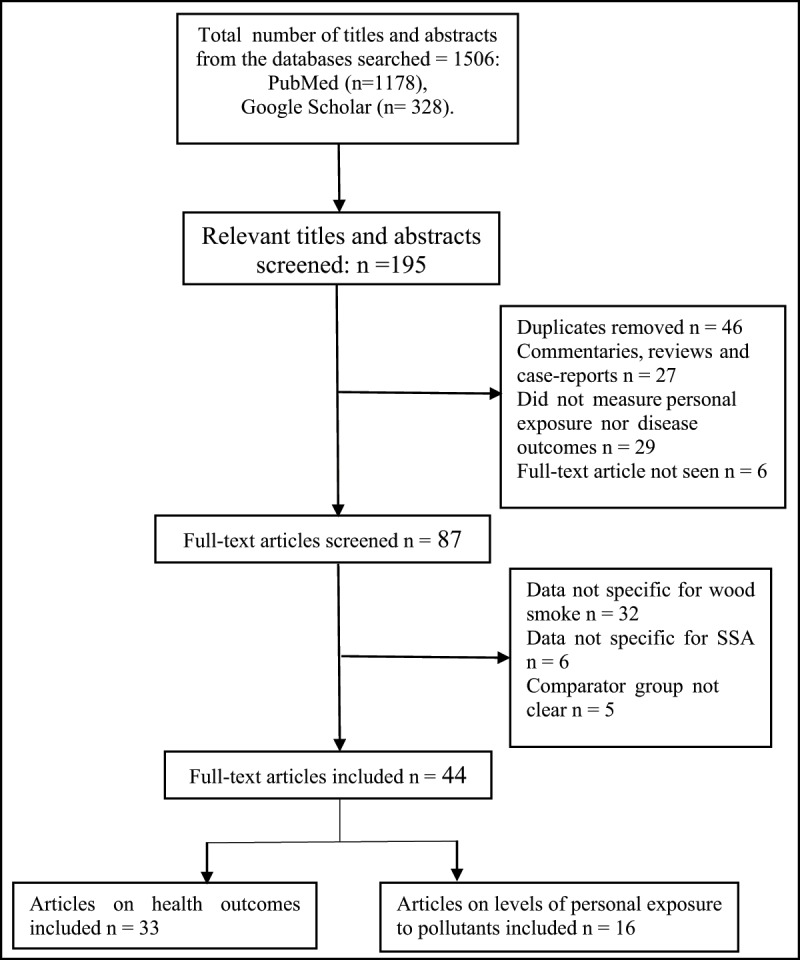
Flow chart of study search and selection process.

The search and selection process for papers included in this review is summarized in Figure 1. From the 1506 abstracts identified from the two databases searched, 195 relevant abstracts were identified. Of these, 44 (forty-four) studies met the inclusion criteria and were included in this review. The characteristics of the included studies are presented in Tables 1 and 2. Sixteen papers reported on levels of exposure to specific pollutants in wood smoke (Table 1), while thirty-three studies reported on health effects associated with wood smoke exposure (Table 2). Two papers [33,34] used the same data but analysed the findings from different viewpoints and so, were merged as one. Five studies [33,34,35,36,37,38] reported both personal exposure and health outcomes of wood smoke exposure and these were reported separately in different tables of this review. The included studies were carried out in fifteen out of the forty-nine countries in sub-Saharan Africa: Nigeria had the highest number of studies (n = 11); followed by Ghana (n = 5); and Kenya (n = 5). Ethiopia, Malawi, Tanzania and Uganda had three studies each; Cameroon and Gambia had two studies each while Burkina-Faso, Congo, Mozambique, South Africa, Sierra Leone and Zambia had one study each. One study [39] combined data from demographic health studies conducted in 23 countries in sub-Saharan Africa. The population groups studied were mainly women (20 studies) and children under five years of age (7 studies). Most of the studies (n = 13) were carried out in rural areas. The study design used in the reviewed papers were mostly cross-sectional design (n = 32). Case-control designs (n = 7), longitudinal cohort (n = 2) and randomized controlled trial (n = 3), designs were also used. Studies made comparisons between wood fuels (firewood and charcoal) and LPG/electricity, biolite, ethanol. However, some studies made comparison between firewood and charcoal users, firewood/charcoal users and non-solid fuel users. Two studies compared level of exposure to wood smoke pollutants between traditional wood stoves and improved wood stoves [40,41].

**Table 1:** Summary of epidemiologic studies on levels of exposures to wood smoke pollutants in sub-Saharan Africa.

Reference (Country)	Study design (Setting)	Study Population (Sample size)	Mean ± SD (Range)	Determinants for higher exposure

Personal PM_10_ Exposure				

**Ellegard [35]** **(Maputo, Mozambique)**	CSS (suburb)	Women who are household cooks (Wood users = 114 Charcoal users = 78 Electricity users = 8 LPG users = 3)	Wood: 1200 ± 131 μg/m^3^ Charcoal: 540 ± 80 μg/m^3^ Electricity: 380 ± 94 μg/m^3^ LPG: 200 ± 110 μg/m^3^	Wood users were exposed to higher PM_10_ than charcoal and electricity/LPG users
Ezzati et al. [33,34]* (Kenya)	LCS (Rural).	55 households, 345 Individuals divided into groups by age and sex:		Females within the age of 6–15 and 16–50 had significantly higher exposure than their male counterparts.
		0–5 years Females (n = 52) Males (n = 41)	0–5 years Females: 1317 (1188)^a^ Males: 1449 (1067)^a^	
		6–15 years: Females (n = 61) Males (n = 48)	6–15 years: Females: 2795 (2069)^a^ Males: 1128 (638)^a^	
		16–50 years: Females (n = 65) Males (n = 55)	16–50 years Females: 4898 (3663)^a^ Males: 1018 (984)^a^	
		>50 years: Females (n = 15) Males (n = 8)	>50 years Females: 2639 (2501)^a^ Males: 2169 (977)^a^	
**Personal PM_2.5_ Exposure**				

**Titcombe and Simcik [43]** **(Tanzania)**	CSS	Women Open firewood users (n = 3) charcoal users (n = 3)	Open firewood users: 1574 ± 287 μg/m^3^ Charcoal users: 588 ± 347 μg/m^3^ LPG users: 14 ng/m^3^	Open firewood users were exposed to higher PM_2.5_ than charcoal users.
**Dionisio et al. [49]** **(Gambia).**	CSS (urban, peri-urban and rural areas)	Children under 5 years of age (n = 31)	65 ± 41 μg/m^3^	NR
**Van Vliet et al. [45]** **(Ghana)**	CSS. (Rural)	29 households cooks (95% females)	24-hour average PM_2.5_ Real time 208 (30–618) μg/m^3^	Personal (Integrated) PM_2.5_ and Black carbon were higher for firewood users [(141.9 (112.5, 171.3)^b^; and (9.7 (8.4, 11.1)^b^, respectively], than Charcoal users [(44.6 (1.8, 87.4)^b^ and (3.2 (0.6, 5.8)^b^, respectively].
			PM_2.5_ Integrated 128.5 (16.6–364.6) μg/m^3^	
			Black carbon 8.8 (1.9–18.2) μg/m^3^	
**Downward et al. [44]** **(Ethiopia)**	CCSS (Urban)	Workers in biomass and electricity using bakeries (N = 15 workers per group)	PM_2.5_: Biomass bakers: 430 (2.0)^c^ μg/m^3^ Electric bakers: 216 (2.2)^c^ μg/m^3^	Occupational exposure to biomass smoke in bakery; Number of stoves in use; Additional biomass usage in coffee brewing
			Black Carbon: Biomass bakers: 67 (1.9)^c^ μg/m^3^ Electric bakers: 15 (1.8)^c^ μg/^m3^	
**Okello et al. [14]** **(Uganda)**	CSS (Rural)	General Population (N = 102) divided into 6 age groups. Infants: (n = 17) Young Males: (n = 16) Young females: (n = 17) Adult males: (n = 17) Adult females: (n = 19) Elders: (n = 18)	Infants: 80.2 (1.34)^c^ μg/m^3^ Young males: 26.3 (1.48)^c^ μg/m^3^ Young females: 117.6 (1.49)^c^ μg/m^3^ Adult males: 32.3 (1.97)^c^ μg/m^3^ Adult females: 177.2 (1.61)^c^ μg/m^3^ Elders: 63.9 (2.03)^c^ μg/m^3^.	Women and girls had higher exposure to wood smoke than men and boys.
**Personal CO Exposure**				

**Dionisio et al. [47]** **(Gambia).**	CSS (urban, peri-urban and rural areas)	Children Under 5 years of age (N = 1181)	1.04 ± 1.46 ppm	Rainy season Use of charcoal
**Ochieng et al. [40]** **(Kenya)**	CSS (Rural)	Women Traditional wood stove users: (n = 50) Improved wood stove users: (n = 50)	Traditional wood stove users: 5.12 ± 3.89 ppm Improved wood stove users: 3.72 ± 3.74 ppm	NR
**Yamamoto et al. [48**] **(Burkina Faso)**	CSS (semi-urban)	Women aged 15–45 years and children ≤9 years (N = 148)	Wood users: 3.3 (2.8-3.8)^b^ ppm Charcoal users: 3.3 (2.8–3.7)^b^ ppm	Cooking outdoors was negatively associated with personal CO levels (>2.5 ppm)
**Quinn et al. [36]** **(Ghana)**	CSS (Rural)	Pregnant women enrolled in GRAPHS who were primary cooks in households (n = 1183)	1.6 (1.31) ppm (0.039–15.4 ppm)	NR
**Quinn et al. [37]** **(Ghana)**	RCT (Rural)	Pregnant women enrolled in GRAPHS who were primary cooks in households (N = 35). Randomized to: firewood (n =18) LPG (n =13) Biolite (n = 4)	Pre-intervention CO levels Firewood: 1.04 ppm LPG: 1.74 ppm Biolite: 1.43 ppm Post-intervention CO levels Firewood: 1.55 ppm LPG: 0.63 ppm Biolite: 1.45	Only the LPG group showed a significant reduction in mean CO level following the intervention
**Downward et al. [44]** **(Ethiopia)**	CCSS	Workers in biomass and electricity using bakeries (N = 15 workers per group)	CO: Biomass bakers: 22 (2.4)^d^ ppm Electric bakers: 1 (5.0)^d^ ppm	Occupational exposure to biomass smoke in bakery; Number of stove in use; Additional biomass usage in coffee brewing were associated with higher exposure.
**Yip et al. [41]** **(Kenya)**	Cross-over study (Rural)	Women (N = 237) and children aged <5 years (n = 239)	All women: 1.3 (1.3, 1.4)^d^ ppm TCS Women: 2.2 (1.7, 2.8)^d^ ppm Children: 0.8 (0.7, 0.9)^d^ ppm ICS Women:1.1 (1.0, 1.3)^d^ ppm Children: 0.8 (0.7, 0.8)^d^ ppm	There was a 44.9% reduction in mean personal CO level among women who crossed over to ICS
**Okello et al. [14]** **(Uganda)**	CSS (Rural)	General Population (N = 102) divided into 6 age groups. Infants: (n = 17) Young Males: (n = 16) Young females: (n = 17) Adult males: (n = 17) Adult females: (n = 19) Elders: (n = 18)	CO: Infants: 0.64 (2.12)^d^ ppm Young males: 0.02 (3.67)^d^ ppm Young females: 0.81 (3.83)^d^ ppm Adult males: 0.17 (4.34)^d^ ppm Adult females: 0.95 (3.26)^d^ ppm Elders: 0.54 (3.07)^d^ ppm	Women and girls had higher exposure to wood smoke than men and boys.
**Carboxy- hemoglobin (COHb)**				

**Olujimi et al. [17]** **(Nigeria)**	CCSS (Occupational exposure in a rural setting)	Charcoal workers and Non-Charcoal workers (n = 298 per group)	Carboxyhemoglobin: Charcoal workers: 13.28 ± 3.91 % (5.00–20.00%). Non-charcoal workers: 8.50 ± 3.68% (1.00–18.00%).	Working in a charcoal production site was associated with higher COHb level.
**Polycyclic aromatic hydrocarbons (PAHs)**				

**Titcombe and Simcik [43]** **(Tanzania)**	CSS	Women Open firewood users (n = 3) charcoal users (n = 3)	Total PAH Open firewood users: 5040 ± 909 ng/m^3^ Charcoal users: 334 ± 57 ng/m^3^ LPG: <1 ng/m^3^ Benzo(a)pyrene Open firewood users: 767 ng/m^3^ Charcoal users: 44 ng/m^3^ LPG users: 0 ng/^m3^	Open firewood users were exposed to higher PM_2.5,_ PAH and benzo(a)pyrene than charcoal.
**Awopeju et al. [38]** **(Ile-Ife, Nigeria)**	CCSS (NS)	Women >20 years. Those who worked as street cooks for more than 6 months (n = 188); those who have never been street cooks (n = 197)	Benzene concentration in passive samplers worn by the women. Street cooks: 119.3 (82.7–343.7)^e^ μg/m^3^ Non-street cooks: 0.0 (0.0–51.2)^e^ μg/m^3^, p < 0.001).	Benzene concentration in passive samplers worn by the women street cooks was significantly higher that worn by the controls.
**Olujimi et al. [18]** **(Nigeria)**	CCSS (Occupational exposure in a rural setting)	Charcoal workers at two locations (Igbo-Ora: n = 25; Alabata: n = 20) and Non-Charcoal workers (n = 23)	Urinary1-Hydroxypyrene Charcoal workers: Igbo-Ora: 2.22 ± 1.27 μmol/mol creatinine Alabata:1.32 ± 0.65 μmol/mol creatinine Non-charcoal workers: 0.32 ± 0.26 μmol/mol creatinine	Working in a charcoal production site was associated with higher levels (>0.49 μmol/mol creatinine) of Urinary1-Hydroxypyrene. (RR: 3.14, 95% CI: 1.7–5.8, P < 0.01)

^a^ = Mean (Variance); ^b^ = Mean (95% CI); ^c^ = Geometric mean (geometric standard deviation); ^d^ = Geometric mean (95% CI); ^e^ = median (inter-quartile range); CCSS = Comparative Cross-Sectional Study; CSS = Cross-sectional study; RCT = Randomized controlled trial; NR = Not Reported; GRAPHS = Ghana Randomized Air Pollution and Health Study; CO = Carbon monoxide; * = Personal PM_10_ exposure estimated from a 210, 14-hour days of continuous real-time monitoring of PM_10_ concentration and time-activity budget of the household members.

**Table 2:** Summary of epidemiological studies on health effects of wood smoke in sub-Saharan Africa.

Reference (Country)	Design (Exposure setting)	Population (sample size)	Source of wood smoke (Comparison)	Method of Exposure assessment	Outcome(s) (Method of Assessment)	Health effect (Risk estimate)	Quality score

Respiratory outcomes							

**Ellegard [35]** **(Maputo Mozambique)**	CSS (suburban)	Women who are household cooks (n = 1200)	Firewood and charcoal for domestic cooking (Electricity/LPG users)	Interview: principal fuel, defined as the fuel used most often to cook the main meal; measurement of personal PM_10_.	Cough, Dyspnea, Wheezing, Inhalation and exhalation difficulties (questionnaire); PEFR (mini-wright peak flow meter	Wood users had a significantly higher cough index (2.42 ± 0.104) and lower PEF rates (365 ± 3.4 L/min) than charcoal users (cough index: 1.77 ± 0.108; PEF rates: 382 ± 4.0 L/min) and users of modern fuels (cough index: 1.75 ± 0.198; PEF rates: 379 ± 7.4 L/min). There were no significant differences between the fuel user groups with respect to the non-cough respiratory symptom index.	6
**Ezziati and Kammen [33,34]** **(Laikipia, Kenya)**	LCS (Rural)	55 households made up of Infants (n = 93), Individuals aged 5–49 years (n = 229), Individuals aged >50 years (n = 23).	Firewood and charcoal for domestic cooking (PM_10_ ≤ 1000 vs. PM_10_ > 1000 μg/m^3^); (PM_10_ ≤ 200 vs. PM_10_ > 200 μg/m^3^).	Personal exposures to PM_10_ calculated from Indoor PM_10_ measurement data and time activity budget.	ARI, ALRI (WHO protocols for clinical diagnosis of ARI).	Risk of ARI and ALRI increased with higher PM_10_ exposure. For example, with reference to PM_10_ of <200, the adjusted odds ratio for ARI was 2·42 (1·53–3·83) in children <5 years with PM_10_ 200–500.	7
**Ibhazehiebo et al. [74]** **(Edo, Nigeria)**	CSS (Rural and urban)	Women aged 20–70 years. Active wood users (n = 350); non-wood users (n = 300)	Wood use for cooking. (non-wood users)	Questionnaire: number of years of use of wood as cooking fuel; number of times of such cooking per day.	PEFR (mini-wright peak flow meter), Respiratory symptoms (cough with sputum production, dyspnea, wheezing, chest tightness and chest pain)	Respiratory symptoms were markedly elevated in the subjects compared to controls. Mean PEFR value for the wood users (289 ± 19.6 L/min) was significantly lower than non-wood users (364 ± 17.2 L/min), P < 0.05. PEFR decreased with increase in years of exposure to wood smoke.	3
**Kilabuko et al. [51]** **(Bagamoyo, Tanzania)**	CSS (Rural)	100 households	Wood use for cooking. (Regular women cooks and children under age 5 Vs unexposed men and non-regular women cooks)	Observation; Kitchen, living room and outdoor measurement of PM_10,_ CO and NO_2_.	ARI (questionnaire)	The risk of having ARI was higher for cooks and children under age 5 (exposed group) than the unexposed group (OR: 5.5; 95% CI: 3.6–8.5)	3
**Fullerton et al. [57]** **Malawi**	CSS (Rural and urban areas)	Adults (n = 374)	Firewood vs. Charcoal users	Questionnaire: type of biomass fuel used for cooking	Lung function (spirometry).	Wood users had significantly worse lung function than charcoal users {FEV_1_, ml: 2430 (670) Vs 2780 (680) P < 0.001; Percent predicted FEV_1_: 99 Vs 106, P < 0.008; FVC, ml: 3190 (830) Vs 3490 (870); FEV_1_/FVC ratio: 76.54 (9.11) Vs 79.80 (7.48) P = 0.001} for firewood and charcoal users, respectively.	5
**Ekaru et al. [52]** **(Moi, Kenya)**	Hospital-based CSS (NS)	Children Aged 0–5 years (n = 181)	Use of firewood/charcoal for domestic cooking (Use vs. non-use of firewood/charcoal)	Care-giver reported use of firewood and charcoal for cooking	Pneumonia (clinical diagnosis)	Use of firewood/charcoal were risk factors for mild pneumonia (OR 4.23, CI 3.9–4.6) and severe pneumonia (OR 1.1, CI 1.02–1.26).	4
**Taylor and Nakai** **(Sierra Leone)**	CSS (rural and peri-urban)	Women aged 15–45 years (n = 520); children under 5 years of age (n = 520).	Use of firewood/charcoal for domestic cooking (Wood vs. Charcoal use)	Questionnaire: Type of biomass fuel normally used for cooking	ARI (interview: cough and rapid breath in the last 2 weeks preceding study as proxy for ARI)	Relative to charcoal use, wood use was associated with ARI in children but not in women (adjusted OR = 2.03, 95%CI: 1.31–3.13) and (adjusted OR = 1.14, 95%CI: 0.71–1.82), respectively. ARI prevalence was higher for children in homes with wood stoves (64%) compared with homes with charcoal stoves (44%).	4
**Oloyede et al. [58]** **(Nigeria)**	Comparative CSS (NS)	Children aged 6–16 years. Those living in fishing port (n = 358) and those living in farm settlements (n = 400)	Firewood use in fish drying (Residence vs. Non-residence in fishing port)	Observation: Living in fishing port	Lung function (spirometry).	Children living in fishing port had reduced lung function (FVC: 1.32 ± 0.67; FEV_1_: 1.22 ± 0.62) compared with those living in farm settlements (FVC: 1.45 ± 0.43; FEV_1_: 1.41 ± 0.41. Decline in lung function was associated with increase in duration of exposure to fish drying.	5
**Ibhafidon et al. [59]** **(Ile-Ife, Nigeria)**	CSS (NS)	Male and female Individuals including firewood users (n = 35), kerosene users (n = 34) and LPG users (n = 21).	Firewood for cooking (firewood vs. LPG users)	Modified BMRC questionnaire: predominant cooking fuel	Respiratory symptoms (Modified BMRC questionnaire); lung function (spirometry).	Firewood users reported more respiratory symptoms, compared with LPG users. 95.2% of LPG users had normal lung function (FEV_1_/FVC > 0.7 and predicted FEV_1_ of at least 80%), 71.4% of firewood users, respectively.	4
**Sanya et al. [56]** **(Kampala, Uganda)**	Case. Control study (NS)	Asthma patients aged >13 years. Those with exacerbations (n = 43); Those without exacerbations (n = 43)	Wood and charcoal use for cooking (in-house vs. no in-house wood/charcoal stoves)	Questionnaire: use of in-house wood/charcoal stoves	Asthma exacerbations (clinical assessment)	In-house wood/charcoal use was not associated with increased risk of asthma exacerbations (OR = 0.882, 95% CI: 0.329–2.36, P = 0.802)	4
**Umoh and Peters [20]** **(Akwa-Ibom, Nigeria)**	CCSS (Rural)	Women involved in fish- smoking activities (n = 324) and women involved in fishing but not fish smoking (n = 346)	Wood use in fish smoking (Fish-smokers vs. non fish-smokers)	BMRC Respiratory disease Questionnaire: based report of involvement Fish-smoking	Lung function (spirometry)	Fish smokers had lower lung function (Mean FEV_1_/FVC = 68.8 ± 15.3 %) than non-fish smokers (FEV_1_/FVC = 78.3 ± 9.6 %), P < 0.001.	5
**Ngahane et al. [60]** **(Bafoussam, Cameroon)**	CSS (Semi-rural)	Women >40 years. Those using wood (n = 145) and those using other fuel sources (n = 155) for cooking.	Wood for domestic cooking (wood vs. other fuel [charcoal, gas, electricity] users)	Questionnaire-based report of cooking fuel.	Lung function (spirometry) Respiratory symptoms (questionnaire)	Use of wood as a cooking fuel was associated with impairment of lung function (Adjusted mean difference in FEV_1_= –120; 95% CI: –205, –35; P = 0.005). Prevalence of chronic bronchitis was higher (7.6%) in wood smoke group than the other fuels (0.6%).	3
**Dienye et al. [50]** **(Rivers State, Nigeria)**	CCSS (rural)	Women age ≥15 years. Those involved in fish smoking (n = 210) and those not involved in fish smoking (n = 210)	Wood use for fish smoking (involvement vs. non-involvement in fish smoking)	Self-reported involvement in fish smoking	Respiratory symptoms (questionnaire). PEFR (mini-wright peak flow meter).	Fish smoking was associated with increased risk of sneezing (OR = 2.49, 95% CI: 1.62–3.82; P < 0.001), catarrh (OR = 3.77; 95% CI: 2.44–5.85, P < 0.001), cough (OR = 3.38, 95% CI: 2.22–5.15, P < 0.001) and chest pain (OR = 6.45, 95% CI: 3.22–13.15, P < 0.001). The mean PEFR of 321 ± 58.93 L/min among the fish smokers was significantly lower than 400 ± 42.92 L/min among the controls (p = 0.0001).	4
**PrayGod et al. [54]** **(Mwanza, Tanzania)**	Case-control (NS)	Children. Under 5 years Cases (n = 45), controls (n = 72).	Firewood and charcoal (Firewood/charcoal users vs. Gas/Electricity users); (indoor vs outdoor cooking).	Questionnaire: Source of cooking fuel; location of cook stove.	Clinical/Laboratory diagnosis of pneumonia	Firewood and charcoal use were not associated with increased risk of pneumonia (Unadjusted OR = 2.1; 95% CI: 0.2–27 and OR = 1.9 95% CI: 0.2–18, respectively). An increased risk of severe pneumonia was associated with cooking indoors (OR = 5.5, 95% CI: 1.4–22.1)	5
**Awopeju et al. [38]** **(Ile-Ife, Nigeria)**	CCSS (NS)	Women >20 years. Those who worked as street cooks for more than 6 months (n = 188); those who have never been street cooks (n = 197)	Firewood and charcoal for street cooking (street cooks vs. Non-street cooks.	Questionnaire: number of hour-years of exposure to street cooking; hours spent cooking daily with biomass fuel at home. Quantification of volatile organic compounds (VOCs) in a sub-sample of the women.	Respiratory symptoms (Questionnaire); pulmonary function (spirometry)	The odds of reported cough (adjusted OR: 4.4, 95% CI: 2.2–8.5), phlegm (AOR: 3.9, 95% CI: 1.5–7.3) and airway obstruction, FEV_1_/FVC < 0.7 (adjusted OR of 3.3 (95% CI 1.3 to 8.7) were significantly higher among the street cooks than controls.	5
**North et al. [61]** **(Rural Uganda)**	Prospective cohort study (Rural)	HIV-infected adults aged >18 years (N = 734; 223 males 511 females)	Use of Firewood/charcoal for domestic cooking. (Firewood Vs Charcoal)	Interview: main cooking fuel	cough of ≥4 weeks duration (self-report).	Cooking with firewood was associated with increased risk of chronic cough among females (adjusted OR = 1.41, 95% CI: 1.00–1.99; p = 0.047).	3
**Okwor et al. [21]** **(Ogun, Nigeria)**	CCSS (Rural)	Females aged 13–60 years in two occupations: garri processors (n = 264) and petty traders (n = 264).	Firewood use in garri processing (occupational exposure vs. non-exposure to wood smoke; ≥10years vs <10 years of occupational exposure.	Observation: Working in garri production site	Respiratory symptoms (self-report); pulmonary function (spirometry).	Prevalence of obstructive pulmonary defect (FEV1/FVC < 70%) among the cassava processors was 21.3% compared to 6.4% among petty traders (P < 0.001). Occupational biomass (wood) fuel use (OR = 6.101, 95% CI 3.212–11.590) and working as a cassava processor for ≥ 10 years (OR 14.916, CI 5.077–43.820) were associated with increased risk of obstructive pulmonary defect).	4
**Tazinya et al. [55]** **(Bameda, Cameroon)**	CSS (NS)	Children under 5 years of age (n = 512)	Exposure to firewood smoke >30 minutes/day. non-exposure to firewood smoke	Questionnaire: Exposure to firewood smoke	ARI (WHO guideline for diagnosis management of pneumonia in children	Exposure to wood smoke was associated with ARI (adjusted OR: 1.85, 95% CI: 1.22–2.78).	5
**Cardiovascular outcomes**							

**Alexander et al. [62]^a^.** **(Ibadan, Nigeria)**	RCT (Peri-urban)	Pregnant women (N = 108; randomized to firewood (n = 51); randomized to ethanol (n = 58)	Firewood use in domestic cooking (Firewood vs. Ethanol).	Interview: primary cooking fuel	Blood pressure (standard method).	There was no statistical difference in DBP (model 1: χ^2^_5_ = 5.24; P = 0.39; model 2: χ^2^_3_ = 2:09; P = 0.55) and SBP pressure (model 1: χ^2^_5_ = 4.69; P = 0.45; model 2: χ^2^_3_ = 3.44; P = 0.33) between the women randomized to firewood and those randomized to ethanol.	4
**Quinn et al. [36]** **(Ghana)**	CSS (rural)	Pregnant women enrolled in GRAPHS (n = 817)	Firewood and charcoal use in domestic cooking (1 ppm increase in CO exposure)	72-hour personal CO monitoring	Blood pressure (watch automatic BP monitor).	CO exposure was significantly associated with diastolic blood pressure (each 1ppm increase in exposure to CO was associated with 0.43 mmHg higher DBP [95% CI: 0.01, 0.86] and 0.39 mmHg higher SBP (95% CI: –0.12, 0.90).	7
**Quinn et al. [37]** **(Ghana)**	RCT (rural)	Non- Pregnant women enrolled in GRAPHS (N = 44). Randomized to firewood (n =23), Improved biomass stove (n = 5) and LPG (n =16)	Firewood for domestic cooking. (Firewood vs. Improved biomass stove and LPG)	Observation: use of firewood, improved biomass stoves or LPG stoves for cooking; Personal 72-hour CO exposure monitoring	Ambulatory blood pressure measurement (ABPM)	ABPM revealed that peak CO exposure (defined as ≥4.1 ppm) in the 2 hours prior to BP measurement was associated with elevations in hourly systolic BP (4.3 mmHg [95% CI: 1.1, 7.4]) and diastolic BP (4.5 mmHg [95% CI: 1.9, 7.2]), as compared to BP following lower CO exposures. Use of improved cookstoves was not significantly associated with lower post-intervention SBP and DBP (within-subject change in SBP and DBP of –2.1 mmHg, 95% CI: -6.6, 2.4 and –0.1, 95% CI: –3.2, 3.0, respectively).	7
**Ofori et al. [63]** **(Rivers State, Nigeria)**	CSS (rural)	Women aged 18 and above (n = 389)	Firewood and charcoal (BMF vs. non-BMF)	Questionnaire: predominant cooking fuel	Blood pressure, lipid profile, carotid intima media thickness (all were measured using standard protocols).	Use of BMF was significantly associated with 2.7mmHg higher systolic blood pressure (p = 0.04), 0.04mm higher CIMT (P = 0.048), increased odds of pre-hypertension (OR:1.67, 95% CI: 1.56, 4.99, P = 0.035), but not hypertension OR: 1.23, 95% CI: 0.73, 2.07, P = 0.440)	5
**Reproductive outcomes**							

**Amegah et al. [64]** **Ghana**	CSS (urban)	Mothers and their new born	Charcoal use in domestic cooking. (Charcoal vs. LPG)	Questionnaire: type of cooking fuel used	Birth weight (from hospital records); low birth weight (birth weight ≤2500g)	Exposure to charcoal was associated with reduction in birth weight (adjusted β: –381, 95% CI: –523, –239 and –278, 95% CI: –425, –131 p = 0.000) for all births and term births, respectively. Exposure to charcoal smoke was also associated with increased risk of low birth weight in all births (adjusted RR: 2.41, 95% CI: 1.34, 4.35, P = 0.003), but not term births (adjusted RR: 1.79, 95% CI: 0.81, 3.94, P = 0.112)	6
**Demelash et al. [66]** **(Ethiopia)**	CCS (rural and urban)	Mothers with singleton live birth. Cases: those who gave live births weighing less than 2500g, n =136) controls (those who gave live births weighed more than 2500g (n = 272).	Firewood use in domestic cooking. (Firewood vs. Electricity)	Questionnaire: type of energy used for cooking	Low birth weight (birth weight ≤2500g).	Use of firewood for cooking was associated with low birth weight (Adjusted OR: 2.7; (95% CI:1.00–7.17)	5
**Whitworth et al. [67]** **(South Africa)**	CSS (rural)	Women (n = 420	Firewood use in domestic cooking. (Open wood fires vs. Electricity)	Questionnaire: type of cooking fuel mainly used.	Plasma concentrations of anti-Müllerian hormone (ELISA Method)	Compared with women who used an electric stove, no association was observed among women who cooked indoors over open wood fires (Adjusted β: –3, 95% CI: –22, 21 and –9, 95% CI: –24, 9 for outdoor wood users and indoor wood users, respectively).	5
**Amegah et al. [65]** **Ghana**	CSS (urban and rural)	Women aged 15–49 years (n = 7183)	Use of Biomass fuel (charcoal, firewood and straw/shrubs/grass) for domestic cooking. (biomass fuel vs. Non-biomass fuel (electricity, LPG and natural gas)	Questionnaire: type of fuel used by households for cooking.	Lifetime experience of stillbirth (questionnaire).	Biomass fuel use mediated 17.7% of the observed effects of low maternal educational attainment on lifetime stillbirth risk	3
**Cancer outcomes**							

**Patel et al. [68]** **(Kenya)**	CCS (NS)	Cases: patients with oesophageal squamous cell carcinoma (n = 159); Controls: Healthy individuals without oesophageal squamous cell carcinoma (n = 159).	Firewood and charcoal use in domestic cooking. [Other fuels (kerosene electricity LPG)]	Questionnaire: type of cooking fuel	Oesophageal squamous cell carcinoma (histological diagnosis)	Cooking with firewood or charcoal was independently associated with Oesophageal cancer (OR: 2.32, 95% CI: 1.41–3.84)	4
**Kayamba et al. [69]** **(Lusaka, Zambia)**	CCS (Rural and urban)	Cases: patients with oesophageal squamous cell carcinoma (n = 77); Controls: Healthy individuals without oesophageal squamous cell carcinoma (n = 145).	Firewood and charcoal use in domestic cooking. (non-use of Firewood/charcoal)	Questionnaire: type of cooking fuel	Oesophageal squamous cell carcinoma (histological diagnosis)	Cooking with firewood or charcoal increased the risk of developing Oesophageal squamous cell carcinoma (adjusted odds ratio: 3.5, 95% CI: 1.4–9.3)	5
**Mlombe et al. [70]** **Malawi**	CCS (NS)	Cases: Adult patients with oesophageal squamous cell carcinoma (n = 96); Controls: Healthy individuals without oesophageal squamous cell carcinoma (n = 180).	Firewood/charcoal use in domestic cooking. (Firewood vs. Charcoal)	Questionnaire: type of cooking fuel	Oesophageal squamous cell carcinoma (histological diagnosis)	The odds of oesophageal cancer were 12.6 times higher among those who used firewood compared to those who used charcoal (Adjusted OR: 12.6, 95% CI: 4.2–37.7).	5
**Other health outcomes**							

**Das et al. [73**] **(Malawi)**	CSS (Rural and urban)	Household cooks (n = 655)	Use of firewood, charcoal and crop residue for domestic cooking. (Firewood or crop residue vs. Charcoal)	Interview: cooking technologies and fuels used.	Five categories of health outcomes: cardiopulmonary, respiratory, neurologic, eye health, and burns. (questionnaire).	Use of low quality firewood or crop residue was associated with significantly higher odds of shortness of breath at rest (Adjusted OR: 1.02; 95% CI: 0.24–4.32 and 6.22; 95% CI: 1.13–34.17, respectively), chest pains (Adjusted OR: 3.00; 95% CI: 0.91–9.86 and 4.46; 95% CI: 0.92–21.66, respectively), night phlegm (Adjusted OR: 5.39; 95% CI: 1.06–27.45 and 11.59; 95% CI: 1.38–97.31, respectively), forgetfulness (Adjusted OR: 4.48; 95% CI: 1.42–14.19 and 9.13; 95% CI: 1.86–44.95, respectively) and dry irritated eyes (Adjusted OR: 1.64; 95% CI: 0.54–4.99 and 3.75; 95% CI: 0.81–36, respectively).	4
**Owili et al. [39]** **(23 Countries in SSA)**	CSS	Children under 5 years of age (n = 783,691)	Use of Charcoal as cooking fuel. (Charcoal vs. Clean fuels [electricity, natural gas, biogas or liquefied petroleum gas]).	Questionnaire: type of cooking fuel	All-cause under-five mortality	Use of charcoal as cooking fuel was significantly associated with the risk of under-five mortality in SSA before and after controlling for other indicators. (Crude HR: 1.97, 95% CI: 1.80, 2.16; Adjusted HR: 1.21, 95% CI: 1.10, 1.34).	3
**Belachew et al. [72]** **Ethiopia**	CSS (NS)	The entire population mate (N = 3405); male: 1567 Female: 1838	Use of charcoal for domestic cooking. (Charcoal use vs. No charcoal use)	Questionnaire/observation: charcoal use for cooking	Sick-building syndrome (questionnaire)	Sick-building syndrome was significantly associated with charcoal use as cooking energy source (Adjusted OR: 1.4, 95% CI: 1.02–1.91)	3
**Mbuyi-Musanzayi et al. [71]** **(Lubumbashi, Congo)**	CCS (NS)	Cases: New born with Non-syndromic cleft lip and/or cleft palate (n = 162) Controls: clinically normal newborn (n = 162)	Use of Charcoal as cooking fuel. (Indoor Vs No indoor cooking with charcoal)	Questionnaire: inhouse cooking with charcoal (yes or no)	Non-syndromic cleft lip and/or cleft palate (clinical diagnosis)	Indoor cooking with charcoal was significantly associated with non-syndromic cleft lip and/or cleft palate (OR = 6.536, 95% CI: 1.229, 34.48. p = 0.0001)	3

CSS = Cross-sectional study; CCSS = Comparative cross-sectional study; LCS = Longitudinal cohort study; CCS = Case-control study; RCT = Randomized controlled trial; NS = Not stated; ISAAC = International Study of Asthma and Allergies in Childhood; BMRD = British Medical Research Council; PEFR = Peak Expiratory Flow Rate; GRAPHS = Ghana Randomized Air Pollution and Health Study; ^a^ = only data for baseline firewood users were included in this review.

The outcomes reported in the studies were classified into two: personal exposure levels (Table 1) and health outcomes (Table 2). We identified 16 studies that reported either personal exposure levels to specific pollutants in wood smoke using personal monitors attached to individual clothing, or concentrations of biomarkers of wood smoke in blood or urine. These included personal exposure to PM_10_, PM_2.5_, CO, black carbon, levels of carboxy-hemoglobin, total PAH, benzene, benzo(a)pyrene and urinary1-Hydroxypyrene. Health outcomes reported in the reviewed studies included respiratory illness, cardiovascular, reproductive/pregnancy outcomes, cancer, mortality, non-syndromic cleft lip and/or cleft palate and sick building syndrome.

### Personal Exposure Levels to Pollutants in Wood Smoke in SSA Population

#### Exposure to particulate matter

Personal PM_10_ exposure from household use of wood fuels was reported in two studies [33,34,35]. Higher levels of PM_10_ exposure was recorded for wood (1200 ± 131 μg/m^3^) than charcoal (540 ± 80 μg/m^3^), electricity (380 ± 94 μg/m^3^) and LPG users (200 ± 110 μg/m^3^), respectively. The mean personal PM_10_ exposure levels reported for all categories of fuel users were above the WHO [42] Air Quality Guideline of 50 μg/m^3^ for 24-hour mean PM_10_ exposure [35]. Ezzati and Kammen [33,34], estimated personal exposure to PM_10_ from data obtained from a 210, 14-hour days continuous real-time monitoring of PM_10_ and time-activity budget of household members. In their report, only 20 (6.2%) of the study population had PM_10_ level <200 μg/m^3^ and the mean PM_10_ levels for all the sub-groups were above the WHO AQG.

Personal exposure to PM_2.5_ was measured in five studies. Four of these studies [14,43,45,49] reported exposure from household use of wood fuels. The reported mean personal PM_2.5_ ranged from 26.3 ± 1.48 μg/m^3^ among young males [14] to 1574 ± 287 μg/m^3^ among women firewood users [43]. All the reported mean personal PM_2.5_ levels in the studies were higher than the WHO AQG of 25 μg/m^3^ [42]. Two of the studies [43,45] compared personal PM_2.5_ exposure between firewood and charcoal users. Both studies reported higher level of personal PM_2.5_ in firewood (1574 ± 287 μg/m^3^ and 141.9 μg/m^3^, respectively) than charcoal users (588 ± 347 μg/m^3^ and 44.6, respectively). Mean PM_2.5_ exposure was higher in women (177.2 ± 1.61 μg/m^3^) and girls (177.6 ± 1.49 μg/m^3^) compared to other age groups [14].

Downward et al. [44] recorded high level of PM_2.5_ exposure among bakers exposed to wood smoke in bakery (430 ± 2 μg/m^3^), compared to those using electric cookstoves (216 ± 2.2 μg/m^3^).

Black carbon levels were measured in two studies [44,45]. Van Vliet et al. [45] reported higher mean black carbon level in firewood users (9.7 μg/m^3^) than charcoal user (3.2 μg/m^3^). Compared with individuals working in bakeries that use electric cookstoves (15 ± 1.8 μg/m^3^), those that worked in wood-using bakeries were exposed to higher levels (67 ± 1.9 μg/m^3^) of black carbon [44].

#### Exposure to Carbon Monoxide

Eight studies reported average 24-hour or 48-hour personal carbon monoxide (CO) exposure levels. In all but one study [44], the reported personal CO exposures were below the WHO [46] AQG of 7 μg/m^3^ (6.11 ppm). The reported levels of CO exposure from household use of wood fuels ranged from 0.02 ± 3.67 ppm in young males [14] to 5.12 ± 3.89 ppm among women using traditional cook stoves [40]. Five of the studies [14,36,37,40,41] gave report on CO exposure in women. The reported mean personal CO levels ranged from 0.81 ± 3.83 ppm in young females [14] to 5.12 ± 3.89 ppm in women using traditional wood stove [40]. Three studies [14,41,47] measured CO exposure among children <5 years of age. The reported levels of CO in these three studies were similar and ranged from 0.64 ± 2.12ppm [14] to 1.04 ± 1.46ppm [47]. Two studies [41,48] reported CO levels in both women and children. Okello et al. [14] reported 24-hour CO exposure in six sub-groups of the study population. In their report, women and girls had higher exposure to CO (0.95 ± 3.26 and 0.81 ± 3.83 ppm, respectively) than men and boys (0.17 ± 4.34 and 0.02 ± 3.67, respectively). Downward et al. [44] measured personal CO exposures in bakery workers in Ethiopia and compared CO exposures between bakers who used electric and biomass cookstoves. In their report, bakers who used biomass cookstoves were exposed to higher level of CO (22 ± 2.4 ppm) compared to those that used electric cookstoves (1 ± 5.0 ppm). Yamamoto et al. [48] reported no significant difference in 24-hour personal CO exposure between women and children living in households where firewood (3.3 ppm) and charcoal (3.3) were used for domestic cooking. Personal CO exposure between traditional and improved woodstoves users were compared in two studies [40,41]. Ochieng et al. [40] reported no significant difference in mean personal CO level among traditional (5.12 ± 3.89 ppm) and improved (3.72 ± 3.74 ppm) woodstoves users, but observed a 24.0% reduction in personal CO concentrations associated with stove replacement. In a cross-over study to identify ICS for use in Kenya, Yip et al. [41] reported significant difference in CO exposure among women and children randomized to the two stove types.

Individuals occupationally exposed to wood smoke in bakery [44] had higher level of CO exposure. Olujimi et al. [17] compared COHb levels between charcoal producers and non-charcoal workers. In their report, subjects exposed to wood smoke in charcoal production had higher level of COHb (13.28 ± 3.91 %) than non-charcoal workers (8.50 ± 3.68%). The reported mean COHb levels in both groups were above the WHO guideline of 2% for COHb [46].

#### Exposure to polycyclic aromatic hydrocarbons (PAHs)

Concentrations of PAHs were measured in three studies [18,38,43]. Titcombe et al. [43] reported mean total PAH and benzo(a)pyrene levels of 5040 ± 909 ng/m^3^ and 767 ng/m^3^, 334 ± 57 ng/m^3^ and 44 ng/m^3^, and <1 ng/m^3^ and 0 ng/m^3^ in open firewood, charcoal and LPG users, respectively. Charcoal production was associated with higher levels (>0.49 μmol/mol creatinine) of urinary1-hydroxypyrene (RR: 3.14, 95% CI: 1.7–5.8, P < 0.01) [18]. Awopeju et al. [38] reported median (interquartile range) benzene concentration of 119.3 (82.7–343.7) and 0.0 (0.0–51.2) in passive samplers worn by street cooks and non-street cooks, respectively.

### Sources of wood smoke exposure in SSA

In all but one study [17], the reported sources of exposure to wood smoke were from the use of wood fuels (firewood and charcoal) for domestic or commercial food preparation or processing. Occupational exposure to wood smoke were reported in seven studies. These include charcoal production [17–18], bakery [44], fish drying [20,50], garri (cassava) processing [21] and street cooking [38].

### Risk factors for higher exposure to wood smoke pollutants in SSA

Twelve studies gave reports on some determinants for higher exposure to wood smoke in SSA (Table 1) and these factors varied between studies. Users of unprocessed firewood had higher exposure to PM_10_ [35] PM_2.5_ [43,45], total PAH and benzo(a)pyrene [43] than charcoal users. Use of charcoal stove was associated with higher level of exposure to CO [47]. Female gender was associated with higher exposure to wood smoke pollutants [14,33,34]. Occupational exposure was associated with higher levels of exposure to pollutants [17,18,38]. Other risk factors identified include rainy season [47], cooking indoors [48], use of multiple wood stoves [44] and use of traditional cook stoves [41].

### Health effects associated with wood smoke exposure in SSA

A total of 33 studies gave report on health effects associated with wood smoke exposure. Of these, 18 studies evaluated effect on respiratory outcomes, 4 studies on cardiovascular outcomes, 3 each on cancer and reproductive outcomes and one each on mortality, sick-building syndrome and non-syndromic cleft lip and/or cleft palate, while one study measured five categories of health outcomes.

#### Respiratory outcomes

The respiratory symptoms evaluated differed in the studies. These symptoms included ARI/ALRI, lung function, asthma, cough, wheezing and dyspnea. Six studies evaluated the impact of wood smoke exposure on acute respiratory infections/acute lower respiratory infections [33,34,51,52,53,54,55]. All of these studies recorded positive associations between exposure to wood smoke and ARI. Ezzati and Kammen [33] reported a positive exposure-response relationship for indoor air pollution and ARI in rural Kenya. Females above five years were reported to be at higher risk of ARI or ALRI than males [34]. Kilabuko et al. [51] recorded higher risk of having ARI among cooks and children under age five compared to men (the unexposed group). With reference to children living in homes with charcoal stoves, Taylor and Nakai [53] reported higher odds of having suffered from ARI in children living in homes with wood stoves. Cooking indoors was associated with increased risk of severe pneumonia [54] but not with asthma exacerbations [56].

All the seven studies on lung function [20,21,38,57,58,59,60] reported positive association between wood smoke exposure and reduced lung function. Three of these studies [20,21,38] reported reduced lung function [20] or higher risk of obstructive pulmonary defect [21,38] in women occupationally exposed to wood smoke. With reference to those who have worked as cassava processors for less than 10 years, working as cassava processor for more than 10 years was associated with higher risk (OR: 14.916, 95% CI: 5.077–43.820) of obstructive pulmonary defect [21].

All the three studies [35,50,61] that examined association between wood smoke exposure and cough reported positive associations. North et al. [61] reported higher risk of chronic cough among HIV-infected females using firewood with reference to those using charcoal as cooking fuel.

#### Cardiovascular Outcomes

Association between wood smoke exposure and cardiovascular outcomes were investigated in four studies [36,37,62,63]. These four studies examined the effect of wood smoke exposure on blood pressure in women. Three of these studies were carried out among pregnant women [36,37,62]. Quinn et al. [36,37] recorded positive associations between wood smoke exposure and increase in blood pressure. In another study [63], use of biomass fuel (firewood and charcoal) was significantly associated with 2.7 mmHg higher systolic blood pressure, 0.04 mm carotid intima media thickness (CIMT) and increased odds of pre-hypertension (OR: 1.67, 95% CI: 1.57–4.99, P = 0.035) but not hypertension (OR:1.23, 95% CI: 0.73–2.07, P = 0.44).

#### Reproductive/pregnancy outcomes

Four studies [64,65,66,67] examined the effect of wood smoke exposure on reproductive/pregnancy outcomes. Compared to users of clean fuels (LPG or electricity) exposure to wood smoke in charcoal and firewood was significantly associated with reduced birth weight [64] and low birth weight [64,66]. Use of biomass fuel (charcoal, firewood and shrubs) was reported to mediate 17.7% of the observed effect of low maternal education on lifetime experience of stillbirth among Ghanaian mothers [65]. Whitworth et al. [67] investigated the effect of wood smoke exposure in open wood fires on plasma concentrations of anti-mullerian hormone. In their report, cooking indoors over open wood fires (with reference to cooking with electricity) was not associated with lower plasma anti-mullerian hormone.

#### Cancer

Three studies investigated the association between wood smoke exposure and oesophageal cancer [68,69,70]. In all the studies, exposure to wood smoke was associated with increased risk of oesophageal cancer.

#### Other health outcomes

Owili et al. [39] reported significant association between charcoal use and all-cause under-five mortality in 23 countries in SSA. The effect of wood smoke exposure on non-syndrome cleft lip/cleft palate and sick-building syndrome were investigated by one study each. Indoor cooking with charcoal was associated with cleft lip or cleft palate [71]. Use of charcoal (with reference to non-use of charcoal) was also associated with sick-building syndrome [72]. Das et al. [73] investigated the effect of firewood (with reference to charcoal) on five categories of health outcomes (cardiopulmonary, respiratory, neurologic, eye health and burns). Compared to charcoal use, use of firewood was associated with higher odds of shortness of breath, difficulty in breathing, chest pains, night phlegm, forgetfulness, dizziness and dry irritated eyes.

With the exception of one study [67], other studies that made comparisons between wood fuels and electricity/LPG users [35,59,64,66], reported positive association between wood fuel use and adverse health effects.

All of the five studies that made comparison between firewood and charcoal use [53,57,61,70,73] reported positive associations between firewood use and health outcomes studied. The effect of indoor woods fuel use (Vs outdoor use) was evaluated in three studies [54,56,71]. While Sanya et al. [56] observed no association between in-house use of wood/charcoal and increased risk of asthma exacerbations, indoor cooking with wood fuels was associated with severe pneumonia [54] and non-syndromic cleft lip/cleft palate [71].

The effect of fuel switch on blood pressure was investigated in two studies [37,62]. In these studies, switch to clean fuels was not associated with significant changes in blood pressure.

## Discussion

This systematic review summarized the reports of published studies assessing levels of exposure to pollutants in wood smoke and the disease outcomes associated with this exposure in population living in sub-Saharan Africa. We identified 16 studies that examined personal exposures to specific wood smoke pollutants or measured biomarkers of wood smoke exposure. However, only three of these studies [17,18,43] measured wood smoke biomarkers in body fluid. We also identified 33 studies that examined associations between exposure to wood smoke and health outcomes in this population. Given the high degree of variability in study designs, type of wood fuel used in households (firewood or charcoal, open fire or improved cook stoves), comparator fuels used (electricity, liquefied petroleum gas, charcoal), population groups examined, exposure settings (rural or urban; occupational or environmental) we did not carry out a meta-analysis of the results. The reported levels of exposure to some pollutants (PM_10_, PM_2.5_ and CO and benzo[a]pyrene) were compared with the World Health Organization’s Air Quality Guidelines (AQGs) for the specific pollutants. The overall assessment of evidence of causality between exposure to wood smoke and disease outcomes reported was based on the strength, consistency and plausibility of the associations.

### Personal exposure levels to wood smoke

The levels of exposure to particulate matter (PM_10_ and PM_2.5_) from wood smoke as reported in reviewed studies indicate that the population in sub-Saharan Africa are exposed to very high levels of this pollutants in wood smoke. The reported personal PM_10_ and PM_2.5_ levels for wood users were all above the World Health Organisation’s Air Quality Guideline of 50 μg/m^3^ and 25 μg/m^3^ for 24-hour mean PM_10_ and PM_2.5_, respectively. Although wood users had higher levels of exposure to PM_10_ and PM_2.5_, the reported PM_10_ levels of 380 ± 94 μg/m^3^ and 200 ± 110 μg/m^3^ among electricity and LPG users, respectively^35^ and PM_2.5_ levels of 216 (2.2) μg/m^3^ among electricity users [44] were of concern, given that these fuels do not emit visible smoke. Although the mean carboxy-hemoglobin concentration of 8.5 ± 3.68% reported among non-charcoal producers in Nigeria [17] was significantly lower than that reported in charcoal producers, the value was still 400% higher than WHO [46], reference level of 2% for carboxy-hemoglobin. This could be expected, given that the comparator group in that study was drawn from the same community as the case group [17]. These reports generally suggest that the general population in sub-Saharan African may be significantly exposed to high background levels of ambient particulate matter and other pollutants in wood smoke or other emission sources. There may not be an “unexposed group” in this population, especially among the rural dwellers. However, because exposure to these pollutants lacks unique clinical symptoms, the magnitude and effects of life-long exposure to wood smoke pollutants through use of wood fuels in SSA may go unrecognised and under-reported.

From the reviewed studies, the reported sources of wood smoke exposure in sub-Saharan Africa were mostly through use of wood fuels in domestic cooking. Occupational exposure to wood smoke was reported in seven studies and included the use of wood fuels in bakery [44] commercial fish drying [20,50], cassava (garri) processing [21], street cooking [38] and charcoal production [17,18]. Generally, in SSA, more than 90% of households (especially those living in rural areas) rely on wood fuels as the only source of energy for all their cooking needs [10]. These fuels are burnt on characteristic unvented open fire stoves, emitting significant quantity of smoke containing health damaging pollutants into the immediate environment. Besides these reported sources, the population in SSA could be exposed to wood smoke through other sources [19,75,76,77]. However, the extent and health effects of exposure to wood smoke from these other sources remains to be studied in this population.

### Health effects of wood smoke in sub-Saharan Africa

The health effects associated with wood smoke exposure in the reviewed studies included respiratory and non-respiratory diseases. We recorded strong and consistent associations between exposure to wood smoke and respiratory diseases such as ARI [33,34,51,53], ALRI [33,34,52,54], reduced lung function [20,21,38,57,58,59,60] and cough [35,50,61]. Wood smoke consists of more than 200 distinct organic compounds [2], many of which have been shown to induce acute or chronic health effects in exposed humans. Of these, fine particles are thought to be the best single indicator of the health impacts [2]. Although studies on health effects of respirable particles have focussed on those derived from fossil fuels, wood smoke particles are usually within the size range (0.02–2.5 μm), thought to be most damaging to human health [2]. PM_2.5_ from wood smoke can penetrate into the deep lung, producing a variety of morphological and biochemical changes [24] and resulting in a range of respiratory diseases. The inflammatory potential of particulate matter has been linked to chronic pulmonary diseases [78]. Based on *in vivo* toxicological studies, wood smoke may also affect pulmonary immune defence mechanisms, with the lung macrophages as a likely target [2].

Strong and consistent associations were recorded between wood smoke exposure and blood pressure [36,37,63]. In line with this observation, Dutta et al. [79] reported higher prevalence of hypertension and pre-hypertension among rural Indian women chronically exposed to biomass fuel during cooking. A number of observational studies have linked exposure biomass fuels to other cardiovascular disease outcomes, including stroke [80] and atherosclerosis [81]. In a group of Chinese women, use of biomass fuel for cooking resulted in a median indoor concentration of PM_2.5_ of 52 μg/m^3^ in summer and 105 μg/m^3^ during the winter and were associated with increases in systolic and diastolic blood pressure [82]. Although not fully understood, the mechanisms proposed to explain the cardiovascular effects of biomass exposure include systemic inflammation, particle-induced oxidative stress, endothelial damage, pro-coagulation and autonomic stimulation [83]. During the past four decades, the highest worldwide blood pressure levels have shifted from high-income countries to low-income countries in south Asia and sub-Saharan Africa [84]. Women in a few countries in sub-Saharan Africa (Niger, Guinea, Malawi, and Mozambique) had the highest levels of mean systolic blood pressure, surpassing 132 mm Hg [84]. However, the contribution of wood smoke exposure to the increasing incidence of high blood pressure and other cardiovascular diseases is yet to be investigated in this population.

Consistent association was also observed between wood smoke exposure and oesophageal cancer [68,69,70]. Reports from SSA [85,86] indicate increasing incidence of oesophageal cancer in this region. Most recently, there has been an increasing attention on the high incidence of oesophageal squamous cell carcinoma throughout the eastern corridor of Africa, extending from Ethiopia to South Africa [87] and a call for multisite investigations into its etiology and to identify targets for primary prevention.

Wood smoke contains several known carcinogens including benzo[a]pyrene and benzene [2]. These PAHs have been widely used as markers for carcinogenic risk levels in epidemiological studies [46,88]. The corresponding concentrations for lifetime exposure to B[*a*]P producing excess lifetime cancer risks of 1/10,000, 1/100,000 and 1/1,000,000 are approximately 1.2, 0.12 and 0.012 ng/m^3^, respectively [46]. Similarly, the concentrations of airborne benzene associated with an excess lifetime risk of 1/10,000, 1/100,000 and 1/1,000,000 are 17, 1.7 and 0.17 μg/m^3^, respectively [46]. The reported concentrations of Benzo(a)pyrene [43] and benzene [38] among wood fuel users may therefore have serious implications for cancer in this population. Our finding therefore, necessitates a closer look at exposure to wood smoke as an important etiologic factor, not only for oesophageal cancer, but for other prevalent cancers in this population.

Exposure to wood smoke was strongly associated with poor pregnancy outcomes [64,65,66]. This observation is in line with that of previous systematic reviews on solid fuel use [30], ambient air pollution [89] and secondhand smoke [90] and risk of adverse pregnancy outcomes. Contrary to these findings, exposure to wood smoke was not associated with elevated risk of intrauterine growth retardation (IUGR) in a population-based birth cohort in British Columbia, Canada [91]. It has been suggested that CO in wood smoke may exert its effects on fetal growth indirectly by reducing the oxygen-carrying capacity of maternal hemoglobin, which could adversely affect oxygen delivery to fetal circulation [92]. Because CO also crosses the placental barrier, the resultant fetal tissue hypoxia has the potential to reduce fetal growth [30]. Although highest in Asia, the burden of IUGR in Africa has been estimated at 20%, with the burden in developing countries rated six times higher than that in developed countries [93]. More studies are needed to understand the role of exposure to wood smoke in these and other adverse reproductive outcomes experienced by this population.

Positive associations were reported for sick building syndrome [72], and non-syndromic cleft lip and/or cleft palate [71]. In a large study involving 783,691 children living in 23 countries in SSA, use of charcoal as cooking fuel was significantly associated with the risk of under-five mortality [39]. However, the number of relevant studies on these outcomes is limited, and more information is needed to confirm these observations.

### Determinants for higher exposure

Several risk factors for higher exposure to wood smoke were identified across the studies. It was demonstrated that young and adult women had higher exposures to wood smoke pollutants than their male counterparts. Although the study population in most of the studies were women and children under five years of age, the reports of the two studies [14,33,34] that examined personal exposure levels to pollutants in different population groups showed that women and girls are exposed to higher levels of these pollutants than men and boys of the same age range. Also, infants in the examined households were exposed to higher levels of these pollutants than men and boys and these exposures were linked to their mothers’ 24-h CO exposure in one study [14]. Household cooking is nearly universally done by women and girls, who often also are responsible for the care of young children. Many women in SSA carry their infants on their back while cooking, exposing the infant to smoke from cooking activities. These two groups generally have the highest exposure levels because they are always nearer the stove during combustion and spend longer time at home than the other age groups. Although exposure-response relationship was examined in only one study [33,34], women and infants who represent the population sub-group with higher exposure in households were reported to have higher risk of ARI compared to men [33,34,51].

Over the last two decades, use of improved wood and charcoal stoves have been introduced in many sub-Saharan African countries and there have been significant improvements in stove designs [94]. Although these are viewed as cost-effective strategy to reduce household air pollution through more efficient combustion of wood fuels and ventilation, the evidence on the effect of these improvements on exposure to pollutants has been mixed [95,96,97,98]. While none of the reviewed studies measured personal particulate matter exposure between traditional and improved wood stove users, the reports of two studies comparing personal CO exposure levels between traditional open firewood stove and improved stove users, are inconsistent [40,41]. While Yamamoto et al. [48] reported no significant difference in personal CO exposure between firewood and charcoal users, the levels of personal exposure to PM_10_ and PM_2.5_ in charcoal users [35,43] were lower than that reported for open firewood users, although this remained many folds higher than the WHO AQG for these pollutants. Comparing pollutant emissions in three improved stove types used in sub-Saharan Africa, Mitchell et al. [98] reported that charcoal stoves emitted up to 4 times less PM, but 6 times higher CO than dry wood stoves. These findings suggest that use of these improved wood stoves is still associated with significant exposure to these health damaging pollutants. However, studies that compared health effects between charcoal and unprocessed firewood users [53,57,61,70,73] suggest that use of charcoal (with reference to firewood) may be associated with lower risk of wood smoke-associated diseases.

The impact of fuel switch on personal CO exposure and blood pressure was investigated in two RCT studies [37,62]. The switched from firewood use to LPG use was associated with significant (more than 100%) reduction in personal CO levels among the women [37]. This is in line with the reports of other studies [99]. However, change in CO level did not translate to lowered blood pressure [37] and there was no significant change in blood pressure following switch from firewood use to ethanol [62].

It has been demonstrated that total switch from firewood to biogas as cooking fuel can reduce airborne emissions of PM_2.5_ and CO to levels below the WHO AQG [100], which is likely to produce health benefits associated with reduced exposure to particulate matter and CO in the long run. One major reason that clean fuel interventions have not recorded consistent positive impact on health in this population is that most households only partially convert to the use of these clean fuels and continue to use wood fuels for part of their cooking needs [100]. Also, because particles in ambient air readily penetrate inside residences, individuals that do not use wood fuels can be exposed to smoke from neighbouring households that use wood fuels. More studies are however, needed to ascertain the effect of fuel switch on exposure to wood smoke pollutants in SSA population.

### Limitations of Study

Some limitations, however, were observed across the studies. One major limitation is the use of different comparators in the different studies which made adequate comparison of the results impossible. Another limitation observed in these studies was the use of questionnaire and interview approach for exposure assessment. Only four of the studies examined association between disease and measured exposure to wood smoke [33,34,35,36,37]. For the other studies, the approach used to characterize exposure to wood smoke was mainly questionnaires and interviews on household’s primary cooking fuel. This method of exposure assessment does not provide reliable estimates of individual exposure to wood smoke, as it does not give information on magnitude of exposure to wood smoke [101] and so may affect the estimated relationship between exposure and disease outcomes investigated, because the ‘unexposed’ may still be significantly exposed to wood smoke. However, the approach is simple; use of a particular fuel may be more stable over a year than a single measurement of personal exposure or area concentration [7]. Active personal monitors are effective in accurately monitoring personal exposures, but implementing personal monitoring on a large scale is expensive and almost impracticable in resource-poor settings such as those in SSA [101]. Measurements of wood smoke biomarkers (which represent the absorbed dose of pollutants, accounting for interindividual differences in absorption, ventilation, and personal behaviours that modify exposure) has several advantages over other exposure assessment techniques [101]. However, these biomarkers were measured in only three of the reviewed studies and these studies did not investigate health effects of these exposures. Furthermore, only a few studies had randomized controlled trial (n = 3) or case-control (n = 7) designs. The majority of the studies had cross-sectional designs in which it was not possible to ascertain a causal and temporal relationship between wood smoke exposure and the health outcomes. These major methodological drawbacks had obvious implications for the quality of the reported evidence. However, the generally consistent results from the different study designs indicate that exposure to wood smoke adversely affects the overall health of this population.

### Recommendations

The findings of this review have important public health implications for people living in SSA. It should be noted that there is considerably high level of awareness of the health implications from the use of wood fuels among SSA women [102,103]. Despite this, these fuels have continued to be in use due to high cost of the alternatives, scarcity of refilling points for alternatives (especially in rural areas) and lack of capital [103]. Given these hindrances, it is likely that domestic use of wood fuels will become even more widespread in this population [12]. As a result, the magnitude of exposure to these wood smoke pollutants may remain the same at least in the near future, and more health effects are likely to be associated with wood smoke exposure. Strategies and interventions aimed at reducing smoke emissions should, therefore, be upregulated. There is need for more vigorous campaigns on the dangers of wood smoke and the need for switching to clean fuels. There is also need for adequate education on ways to reduce exposures to these emissions especially among women and those with occupational exposure to wood smoke. Furthermore, government and international agencies should help by subsidizing the cost of clean fuels and making them available in rural areas. These evidences are mostly from studies conducted in women and children. Further research on the effect of exposure to wood smoke pollutants in other population groups, especially the elderly, is urgently needed. Large epidemiological studies, especially prospective cohort and case-control studies with improved measures of exposure such as active personal monitors and specific wood smoke biomarkers are needed to adequately quantify exposures and estimate their effects on health in this population.

## Conclusion

Available evidence suggests that the general population in sub-Saharan Africa are exposed to very high levels of pollutants in wood smoke from use of wood fuels such as firewood and charcoal for domestic cooking needs and this exposure is associated with a number of disease outcomes, especially respiratory diseases in women and children under five years of age. There is urgent need for effective strategies to reduce exposure in other to control the morbidity and mortality rate attributable to wood smoke exposure in this population.
